# Preliminary investigation of the design space of geared magnetorheological actuators for safer robotic manipulators

**DOI:** 10.3389/frobt.2025.1581651

**Published:** 2025-06-05

**Authors:** Samuel Gingras, Alexandre St-Jean, Jean-Sébastien Plante

**Affiliations:** Department of Mechanical Engineering, Université de Sherbrooke, Sherbrooke, QC, Canada

**Keywords:** robot safety, collaborative robots, magnetorheological clutch, actuator, robot architecture, gearing ratio, physical human-robot interaction

## Abstract

Geared magnetorheological (MR) actuators have the potential to provide safe and fast physical interactions between human and machine due to their low inertia and high bandwidth. The use of MR actuators in collaborative robotics serial manipulators is only emerging and the design space of this approach is unknown. This paper provides a preliminary understanding of this design space by studying how much gearing can be used between the MR actuators and the joint outputs while maintaining adequate safety levels for collaborative tasks. An analytical collision model is derived for a 6 degrees-of-freedom serial manipulator based on the geometry of the well-known UR5e robot. Model validity is confirmed by comparing predictions to experimental collision data from two robots, a UR5e and a MR5 equivalent. The model is then used to study the impact of gearing level on safety during eventual collisions with human. Results show that for both technologies, robot safety is governed by the balance between the reflected mass due to structural mass and actuator rotational inertia. Results show that, for the UR5e geometry studied in this paper, MR actuators have the potential to reduce the reflected mass in collisions by a factor ranging from 2 to 6 while keeping gearing ratios above 100:1. The paper also briefly studies the influence of robot shape on optimal gearing ratios showing that smaller robots with shorter range have lower structural mass and, thus, proportionally benefit even more of MR actuators. Delocalizing wrist actuators to the elbow has a similar impact since it also reduces structural mass. In all, this work suggests that MR actuators have a strong potential to improve the “hapticness” of collaborative robots while maintaining high gearing ratios.

## 1 Introduction

Collaborative robots or “cobots” have recently been introduced in many industrial sectors, such as aerospace (Bluethmann et al., 2003; Bo et al., 2016), automotive (Peshkin et al., 2001), construction (Gambao et al., 2012) and manufacturing (Bernhardt et al., 2008) just to name a few. Regardless of sector, collaborative robots must fundamentally be harmless to humans during eventual collisions by limiting impact forces. To this end, the ISO/TS 15066 Standard on collaborative robots defines collision force limits that cannot be trespassed to guarantee safety. The standard also defines equivalent robot reflected masses at end effector corresponding to these force limits (ISO, 2016).

Despite enthusiasm for collaborative robots, their safe operating speeds remain low in practice and there is a shortfall to achieve the same production rates associated with classic, non-collaborative, industrial robots. For example, FANUC LR Mate series’ robots have a calculated end-effector maximum speed of 11 m/s (FANUC, 2022), while the UR5e cobot from Universal Robots has a typical tool speed of 1 m/s (UniversalRobots, 2024) and even slower when operating in full collaborative mode.

Solutions to improve cobots safe speeds have been proposed and can be categorized either as active or passive systems (Sanneman et al., 2020):

*Active systems* detect human presence before a collision happens. For example, active robot skin can detect electrical changes within a short range around the robot (Ulmen and Cutkosky, 2010). Other active systems can include standard industrial sensors such as light curtains and proximity laser sensors, which allow to adjust the robot speed dynamically according to the proximity of humans (Sanneman et al., 2020; Byner et al., 2019).

*Passive systems* focus on reducing collision damage by design, that is, by introducing compliant elements or by reducing robot’s reflected mass (Duchaine et al., 2009; Kim et al., 2012). UR robots (UniversalRobots, 2024), Franka Emika’s Panda Arm (FrankaEmika, 2022) and Kuka’s IIWA arm (KUKA, 2022) are examples of cobots implementing passive strategies, mostly by reducing structural mass (Sanneman et al., 2020).

Reducing reflected mass not only implies reducing structural mass, but also requires reducing the reflected mass coming from actuators rotational inertia (García et al., 2020). Lightweight gearing technologies with low inertia have been developed like Harmonic-Drives (HD), known from the UR robots, Planetary Gearheads/Gear train (PGT) and Cycloid Drives. Others like the REFLEX Torque Amplifier, Archimedes Drive, NuGear, Bilateral Drive, Gear Bearing Drive and Galaxie Drive are also in development (García et al., 2020). Another strategy to reduce actuator reflected inertia is to reduce the gearing ratio as much as possible by using high-torque motors leading to Quasi-Direct-Drive or Lightly Geared actuators (Seok et al., 2015; Sensinger et al., 2011). Such designs are used by manufacturers like Genesis Robotics or Halodi Robotics AS (García et al., 2020). Since they operate with minimal gearing ratios in the 10:1 range, lightly geared solutions, however, have limited torque density and a high energy consumption at low speeds due to motor resistive heating (Wensing et al., 2017). Lightly geared actuators perform well for walking and running robots operating at high speeds, but the aforementioned drawbacks become prohibitive for holding fixed positions under load for long periods of time.

Still in passive systems, magnetorheological (MR) actuators have shown potential to reduce actuator reflected inertia to the end-effector by decoupling the inertia coming from the electric motor through a fluidic interface (Fauteux et al., 2010; Shafer and Kermani, 2011; Najmaei et al., 2015; Pisetskiy and Kermani, 2018; Moghani and Kermani, 2020). Early studies revealed that MR actuators used in direct-drive or with low gearing ratios (Chouinard et al., 2018) perform well in force control applications. However, these studies did not consider torque-to-mass ratio as a design metric, leading to impractically heavy actuators. Subsequent development of MR actuators constantly increased the gearing ratio between MR clutches and system output, thus improving compactness and energy efficiency. Most recent MR actuators have shown promising results for vibration control on active seats (Begin et al., 2018), automotive active suspensions (East et al., 2021), haptic control inceptors (Julio et al., 2015), cable-driven robots (Viau et al., 2017; Gervais et al., 2022), supernumerary robotic arms (Véronneau et al., 2019; Denis et al., 2022; Veronneau et al., 2020) and exoskeletons (Khazoom et al., 2020; Khazoom et al., 2019).

**FIGURE 1:**
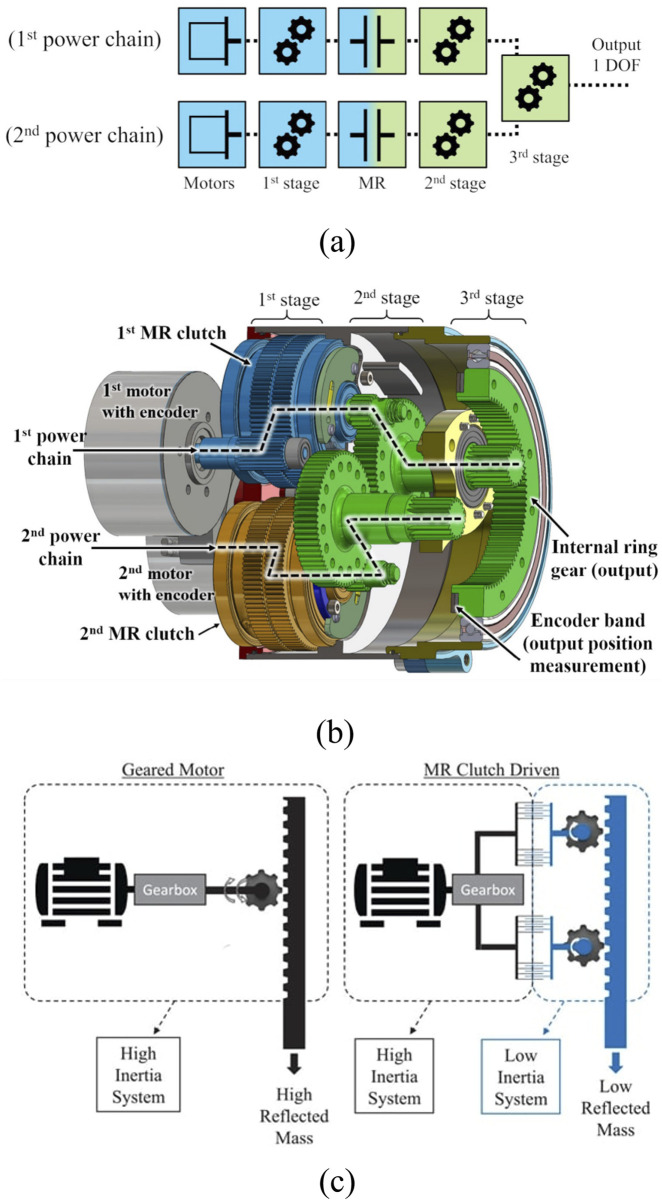
Proposed architecture of MR actuator using two power chains. **(a)** Schematic view of the two power chains, depicting the motors, pre-clutch gearing, MR clutches, actuator gearing and output. **(b)** Detailed view of a MR actuator, showing the motors, MR clutches and gearing. **(c)** Schematic comparison of a geared motor with a high reflected mass at the output and a MR clutch driven actuator with a low reflected mass at the output from its low inertia system (Véronneau et al., 2023).

As illustrated on Figure 1, the latest embodiment of so-called *geared* MR actuators consist of two (or more) independent actuation chains connected to a single output. Each chain follows the sequence of motor–pre-clutch gearing–MR clutch–actuator gearing–output. The key advantage of this approach remains to decouple the inertia of the motor side of the clutches from the actuation side of the clutches when MR clutches are slipping, see Figure 1. MR clutches control the torques of the actuation chains while motor speed controls the slip level of the clutches (Véronneau et al., 2023). The result is a lightweight, low inertia, and high bandwidth actuation system that can be controlled in impedance, even without closing the loop on joint torque. Benchmarking geared MR actuators with respect to current actuation technologies on actuator design metrics such as torque density, power density, force bandwidth, backdriveability, and efficiency to name a few is beyond the scope of this work. First work to that end has been made and shows that, for a same actuator torque, geared MR actuators have a strong potential to lead to better dynamic performance (force bandwidth and backdriving forces) while being lighter than harmonic drives and quasi-direct drive actuators (Plante et al., 2025). Comparison with other actuation alternatives such as serial elastic actuators, pneumatics, and hydraulics has not been considered yet.

Geared MR actuators do have limitations. First, they are mechanically complex since they use two mechanical chains per actuator, doubling the number of components with two motors/drives and two separate gearing chains. Assembling such actuators is time consuming, and must be done with care to minimize assembly errors. Second, geared MR actuators have gears between the MR clutches and actuator output. The imperfections of these gears cannot be filtered out by the fluidic interface and the gearing quality must be high.

St-Jean developed and verified a collision model on a tendon-driven 3 DOF MR robot with actuators delocalized to its base, showing that MR actuators are promising in mechatronic applications such as 3 DOF tendon robots and 6 DOF serial chain configuration, with control strategies exploiting the fast reaction of MR actuators being a key element (St-Jean et al., 2024). However, no such 6 DOF MR robots existed at the time of St-Jean’s study and the design space of geared MR actuators for serial-chain cobots is still unknown. Data is non-existent concerning the interactions between joint size, gearing ratio, performance, and human safety.

This paper provides a first understanding of the design space of geared MR actuators for serial-chain cobot applications by systematically studying the impact of gearing ratio of each actuator in the chain on the safety potential. Understanding the effect of the actuator gearing ratio is key to the maximization of the safe speed of robots. Getting a complete mapping of the full design space is well beyond this work. The aim here is to focus on the main design parameter, that is the gearing ratio, and to identify dominant design trends backed by experimentally validated model predictions. Since this work is a first study, orders-of-magnitude precision levels are judged sufficient.

The paper starts off by developing a theoretical modeling framework predicting the equivalent collision mass of a 6 degrees of freedom (DOF) cobot including the effects of the mass and inertia of geared MR actuators along with gearing ratio. The model is then validated experimentally by performing collisions on two 6 DOF cobots, one powered with MR actuators, and one being a commercial reference product using harmonic drives. This paper is the first to present experimental collision data of a MR-powered 6 DOF serial chain robot. Finally, the model is used to study the effect of gearing ratio on selected cobot configurations and basic design guidelines are drawn. The model uses modeling techniques commonly found in robotics, but here applied in the context of MR actuators.

## 2 Materials and methods

### 2.1 Collision model and ISO/TS 15066 safety standard

The ISO/TS 15066 Standard defines the maximum safe speed of a cobot’s end-effector based on an energy transfer collision model with a human. While the ISO/TS 15066 Standard outlines multiple safety requirements for cobots, this paper focus only on the maximum collision force as it is generally considered as the most restrictive and challenging criteria to satisfy. The model considers that the kinetic energy $E_{k}$ coming from the robot is entirely transferred to a spring-mass system representing the human body part involved in the collision (ISO, 2016).

**FIGURE 2:**
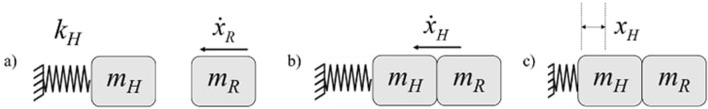
1-D collision model when masses are: **(a)** pre-impact, **(b)** at the instant of impact, and **(c)** post-impact where maximal force is reached. Adapted from St-Jean et al. (2024).

The collision model used in this work is described in St-Jean et al. (2024) and is briefly recalled here for completeness. The model considers rigid bodies and the conservation of momentum of a collision in one dimension, such as shown in Figure 2, where $k_{H}$ is the effective spring constant for specific regions of the human body (e.g., head, hand, …), $x_{H}$ is the maximum deflection, ${\dot{x}}_{R}$ the relative speed between the robot and the specific body region, ${\dot{x}}_{H}$ the post-impact velocity, $m_{H}$ the effective mass of the specific body region, and $m_{R}$ the reflected mass of the robot. For simplicity, this paper considers, as a first approximation, an infinite stiffness for the transmission between the actuator and the link of the robot.

The maximum collision force $F_{\max}$ can thus be expressed by Equation 1:

$$F_{\max}=\sqrt{\frac{k_{H}{\left(m_{R}\right)}^{2}}{m_{R}+m_{H}}}{\dot{x}}_{R}$$ (1)

where $F_{\max}$ , $k_{H}$ and $m_{H}$ are established by ISO/TS 15066 Standards (ISO, 2016). The only remaining variable is the robot reflected mass $m_{R}$ which is the safety metric controlling the robot effector’s maximal safe velocity. A 6 DOF model as presented in the next section is required to theoretically evaluate $m_{R}$ for a given 6 DOF manipulator.

### 2.2 6 DOF cobot dynamic model

A n DOF manipulator’s inertial properties are represented in the joint space by the n -by- n configuration dependent matrix A(q) , often called the Mass matrix or Inertia matrix, where q is the joint angles vector. A(q) is associated to the kinetic energy $E_{k,m}$ of the manipulator, see Equation 2:

$$E_{k,m}=\frac{1}{2}{\dot{q}}^{T}A\left(q\right)\dot{q}$$ (2)

where $\dot{q}$ is the angular speed at joints. The motion equation is defined by Equation 3:

$$A\left(q\right)\ddot{q}+b\left(q,\dot{q}\right)+g\left(q\right)=\Gamma$$ (3)

where $b\left(q,\dot{q}\right)$ is the vector of centrifugal and Coriolis joint forces, g(q) is the gravity joint-force vector and Γ is the vector of generalized joint forces (Khatib, 1995).

An operational coordinate system associated with the base of the robot, which is fixed, is used to find the m -by- m Operational Kinetic energy matrix Λ(x) , where m is the number of independent parameters used to describe the position and orientation of the end-effector in the operational referential and x are the operational coordinates. The relation between A(q) and Λ(x) is defined by Equation 4:

$$\Lambda \left(x\right)=J^{-T}\left(q\right)A\left(q\right)J^{-1}\left(q\right)$$ (4)

involving J(q) , the Jacobian matrix of the manipulator. The operational kinetic energy matrix for a translation movement Λ(v) is determined with Equation 5:

$${\left(\Lambda_{v}\right)}^{-1}=J_{v}\left(q\right)A^{-1}\left(q\right)A\left(q\right)J_{v}^{T}\left(q\right)$$ (5)

where $J_{v}\left(q\right)$ is the manipulator Jacobian associated with the linear velocity at the end-effector. The reflected mass at the end-effector for a translational displacement $m_{u}$ is then defined with Equation 6:

$$\frac{1}{m_{u}}=u^{T}\Lambda_{v}^{-1}u$$ (6)

where u is a directional vector. The same principle can be applied for a rotational task, see Equations 7, 8:

$${\left(\Lambda_{w}\right)}^{-1}=J_{w}\left(q\right)A^{-1}\left(q\right)A\left(q\right)J_{w}^{T}\left(q\right)$$ (7)

$$\frac{1}{I_{u}}=u^{T}\Lambda_{w}^{-1}u$$ (8)

where $\Lambda_{w}$ , $J_{w}\left(q\right)$ and $I_{u}$ are respectively the kinetic energy matrix for a purely rotational task, the manipulator Jacobian associated with the angular speed of the end-effector and the reflected inertia for a rotational displacement at the effector (Khatib, 1995).

A(q) is defined by the sum of the structural inertia matrix $A_{s}$ and the actuator’s inertia matrix $A_{a}$ , see Equation 9 (Lynch and Park, 2016):

$$A\left(q\right)=A_{s}+A_{a}.$$ (9)

This way, A(q) considers the kinetic energy of the moving rigid bodies in space, namely, the robot members and actuators, and the rotational kinetic energy of the moving parts inside the actuators (rotor, gears, etc.) (Wensing et al., 2017). The balance between these two forms of stored energy is key to understand design trends discussed later in the results section.

To determine $A_{s}$ , the method used is based on a manipulator model proposed by K. Kufieta (Kufieta, 2014), where each member is decomposed into cylinders of constant independent density, both for the structure of the member and the associated actuators, to represent the position of the center-of-mass of the member. A change in the actuator mass is represented by a change in its cylinder density, resulting in a shift of the center-of-mass of the associated member. If all members are rigid bodies, Equation 2 becomes Equation 10:

$$E_{k}=\frac{1}{2}{\dot{q}}^{T}\left(\overset{n}{\underset{i=1}{\sum }}\left(m_{i}J_{v,m,i}+J_{w,m,i}^{T}R_{i}I_{m,i}R_{i}^{T}J_{w,m,i}\right)\right)\dot{q}$$ (10)

such that:

$$A_{s}=\overset{n}{\underset{i=1}{\sum }}\left(m_{i}J_{v,m,i}+J_{w,m,i}^{T}R_{i}I_{m,i}R_{i}^{T}J_{w,m,i}\right)$$ (11)

where $J_{v,m,i}$ and $J_{w,m,i}$ are respectively the linear and angular parts of the Jacobian matrix from the robot base to the center-of-mass of the link $L_{i}$ , $R_{i}$ is the rotation matrix from base to link $L_{i}$ , $I_{m,i}$ is the inertia tensor of the link $L_{i}$ expressed in the body attached frame and $m_{i}$ is the mass of the link $L_{i}$ .

$A_{a}$ is defined by the reflected rotational inertia of the actuators. It is a diagonal matrix composed of the reflected inertia at the output of each actuator. For conventional actuators, the reflected inertia at the output is the inertia of the rotor of the motor multiplied by the squared gearing ratio, see Equation 12:

$$A_{a}={\left(\begin{array}{ccc} G_{1}^{2}I_{\mathrm{rotor,1}} & \cdots & 0\\ \vdots & \ddots & \vdots \\ 0 & \cdots & G_{n}^{2}I_{\mathrm{rotor,n}} \end{array}\right)}_{n\times n}$$ (12)

where G are the gear ratios for the actuators and $I_{\mathrm{rotor}}$ are the rotor’s scalar inertias about the rotation axis (Lynch and Park, 2016). For MR actuators, the motor’s rotor is replaced by the clutch output inertia which is multiplied by the subsequent gearing ratio squared since the clutch decouples the inertia of the rotor and the gearing placed before the clutch. Thus, when looking at Equation 12, G becomes the gearing ratio between the clutch and the output and $I_{\mathrm{rotor}}$ the clutch output inertia (Khazoom et al., 2019). It becomes clear that the choice of the gearing ratio while designing an actuator becomes important, as it impacts $A_{s}$ and $A_{a}$ from the actuator mass and reflected inertia at the output.

From Equations 5, 6, 9, if $m_{R}$ is for a translational displacement, $m_{R}$ becomes:

$$\frac{1}{m_{R}}=u^{T}J_{v}{\left(A_{s}+A_{a}\right)}^{-1}J_{v}^{T}u.$$ (13)

Note that Equation 13 has the same form as Equation 9 showing a relation between reflected mass and the sum of the structural and actuator inertia, see Equation 14:

$$m_{R}∼\left(A_{s}+A_{a}\right).$$ (14)

Actuator mass and inertia models are developed hereafter to define $A_{s}$ and $A_{a}$ in terms of actuator’s design parameters and gearing ratio.

### 2.3 Actuator mass and inertia analysis framework

Scaling laws for geared MR actuator’s mass and inertia are only emerging. A first set of scaling laws have been developed for actuator benchmarking purposes (Plante et al., 2025) and are recalled hereafter for completeness of this paper studying the effect of gearing ratio on serial-chain robot safety. Geared MR actuators are a new design paradigm and have no known scaling laws. Therefore, this section develops first order scaling laws for mass and inertia properties that can capture the effect of gearing ratio. These same scaling laws are derived such that they can be used for classic harmonic drive gearmotors taken here as a benchmarking reference.

Geared MR actuator’s mass and inertia properties are derived from experimentally validated parameters from 3 design generations.

1. *Gen1* is the first generation of geared MR actuator prototypes and uses basic, single loadpath spur gearing and drum-type MR clutch technology. The actuator gearing ratio (between clutch and output) is 20.4:1 and the pre-clutch ratio (between motor and clutch) is 4.8:1. Maxon EC45 80W motors are used. *Gen1* actuators have been built and tested (Véronneau et al., 2023).
2. *Gen2* is the second generation of geared MR actuators improving over the *Gen1* by introducing multiple loadpath gearing while keeping well-proven drum clutches. The gearing uses 3 planets epicyclic geartrains with dual output idlers. The actuator gearing ratio is 57:1 and the pre-clutch ratio is 3.96:1. Maxon EC45 80W motors are used. *Gen2* actuators have been built and tested (Plante et al., 2025).
3. *Gen3* is the third generation of geared MR actuators improving over the *Gen2* by introducing low inertia and low friction 3D printed disk clutches while keeping multiple loadpath gearing. The gearing uses the same 57:1 epicyclic gearing of the second generation but with an additional 2:1 stage bringing the total actuator gearing to 114:1. The pre-clutch ratio is 1:1. TQ 5014 frameless brushless motors are used. *Gen3* actuators have not been fabricated yet and represent a realistic design boundary pushing gearing as high as possible toward the upper bounds of the MR technology.

Universal Robot’s UR3 to UR16 cobots are taken here as a gold standard reference. Their actuators are rated from 12 Nm (size 0) to 330 Nm (size 4) and use Kollmorgen KBM series frameless motors combined to 100:1 harmonic-drives (Boscariol et al., 2020). While a comparison with other actuators from other manufacturers would be beneficial, the choice is made to solely keep the UR systems since: (1) the goal of this study is to compare the MR technology with harmonic drives, not to compare variants of harmonic drives amongst themselves; and (2) the UR systems were one of the first commercially available cobot technologies and are still today one of the most popular, making them a robust, well respected, common ground for comparison.

Electric motors are used in both geared MR and UR actuators to generate mechanical power. Their basic parameters consist of continuous torque $T_{m,c}$ , peak torque $T_{m,p}$ , maximum power ${\dot{W}}_{m,p}$ , maximum speed $\omega_{m}$ , and the breaking speed between constant torque mode and constant power mode $\omega_{b}$ .

Motor loading is expressed with Equation 15 by an overload factor $OL_{m}$ defined by the ratio of the actual torque $T_{m}$ to the continuous torque:

$$OL_{m}=\frac{T_{m}}{T_{m,c}}.$$ (15)

The peak-to-continuous torque ratio $\left(T_{m,p}/T_{m,c}\right)$ is taken as the maximum permissible overload factor.

The analysis framework is based on the concept of an actuator’s brake torque $T_{a,brake}$ , that is the measurable torque at the actuator output, and how it relates to the mass and inertia of the actuator. The brake torque is found by subtracting the frictional torque losses $T_{a,friction}$ from the lossless or ideal indicated torque $T_{a,ind}$ , as shown in Equation 16:

$$T_{a,brake}=T_{a,ind}-T_{a,friction}.$$ (16)

The mechanical efficiency $\eta_{a,mech}$ characterizes all frictional torque losses acting on the system and is defined by Equation 17:

$$\eta_{a,mech}=\frac{T_{a,brake}}{T_{a,ind}}.$$ (17)

Only torque destroying mechanisms such as Coulomb and viscous friction in bearings and gears influence the mechanical efficiency. Clutch slippage has no effect on mechanical efficiency since torque is preserved through a slipping clutch. Slippage therefore only influences energy conversion efficiency with a velocity loss during slipping.

The mechanical efficiency of actuator gearing, be it spur, planetary or harmonic, fundamentally depends on gearing ratio and output speed. Given that the gearing ratios considered in this study are in a relatively narrow range (50:1 to 150:1), and that the output speeds are relatively low (around 10 to 30 RPM), a first simplifying assumption is made by using a constant mechanical efficiency value of 0.85 for both harmonic drives and planetary gearing. This value is taken as a global average of manufacturers data, that is conservatively made in favor of harmonic drives whose efficiency drops rapidly, well below 85%, when output speed increases.

**FIGURE 3:**
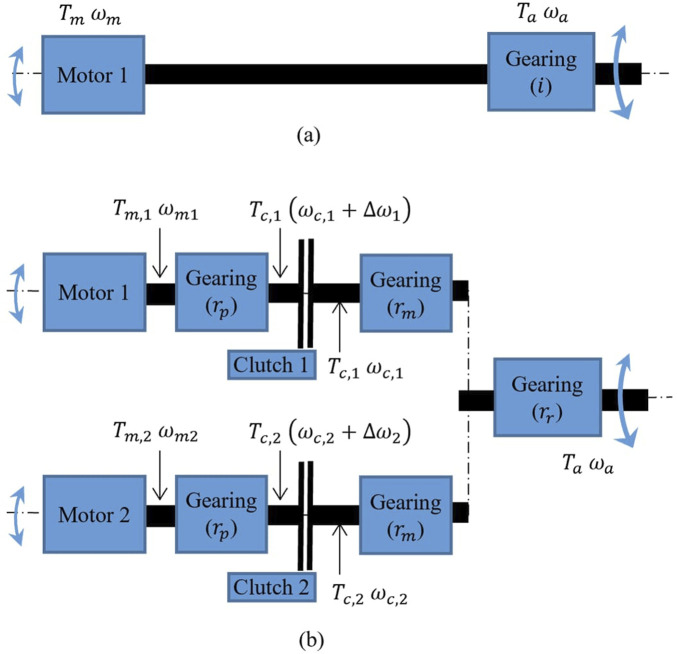
Power flow of **(a)** conventional gearmotor and **(b)** geared MR actuator with 2 actuation chains. Adapted from Plante et al. (2025).

Topologies and power flow of conventional and geared MR actuators are shown in Figure 3. In conventional actuators, see Figure 3a, the indicated output torque is the motor’s torque $T_{m}$ multiplied by the gearing ratio i , as shown in Equation 18:

$$T_{a,ind}=T_{m}i.$$ (18)

In geared MR actuators, unlike most conventional actuators, multiple sub-actuation chains can be used in parallel, see Figure 3b. On the motor side of the clutch, the motor can use a light pre-clutch gearing, $r_{p}$ , typically between 1:1 and 5:1, to better fit actuator performance requirements. On the output side of the clutch, the chains have a main gearing stage, $r_{m}$ , before being merged into a recombination gearing stage, $r_{r}$ , for a total actuator gearing of $i=r_{m}r_{r}$ . i is the gearing ratio governing actuator dynamics since the clutch decouples the dynamics of the motor side of the clutch from the output side of the clutch.

MR fluids in MR clutches behave like Bingham fluids and clutch torque is generated by a magnetically controllable torque, $T_{Y}$ , due to the fluid’s yield stress and a viscous torque, $T_{S}$ , due to fluid’s shear stresses during slippage, see Equation 19 (Avraam, 2009):

$$T_{c}=T_{Y}+T_{S}.$$ (19)

Assuming linear viscosity, the torque due to shear stress is defined by Equation 20:

$$T_{S}=b\Delta \omega$$ (20)

where b is a damping constant with units of Nm/RPM. Both the magnetic and shear stress of MR clutches can be controlled independently, the former by the magnetic field in the fluid and the later by the clutch’s slip speed. For simplicity, this study considers only one control mode called “combined slip control” where the two chains work together, equally sharing the load.

Referring to Figure 3b shaft speeds through the clutches is controlled such that a clutch’s input speed is always higher than its output $\omega_{c,1}+\Delta \omega_{1}\geq \omega_{c,1}$ and $\omega_{c,2}+\Delta \omega_{2}\geq \omega_{c,2}$ . The indicated torque of a MR actuator is the sum of the clutches’ torques, $T_{c}$ , in all actuation chains. If all clutch torques are equal such as supposed here, then the actuator indicated torque is defined by Equation 21:

$$T_{a,ind}=NT_{c}i$$ (21)

where N is the number of actuation chains.

The following subsections develop relationships between an actuator’s *brake torque* and its *mass* and *inertia*, expressed as *Torque-to-mass* and *Torque-to-inertia* ratios, respectively. Note that the analytical development contains many simplifying assumptions that were necessary to obtain closed-form expressions. This is judged acceptable for a preliminary analysis such as intended in this paper.

### 2.4 Actuator torque-to-mass

Geared MR actuators have 6 component groups: motor(s), pre-clutch gearing(s), clutch(s), main gearing(s), recombination gearing, and frame. The total mass $m_{a}$ is the sum of all parts, as shown in Equation 22:

$$m_{a}=N\left(m_{m}+m_{g,p}+m_{c}+m_{g,m}\right)+m_{g,r}+m_{f}$$ (22)

where $m_{m}$ is the motor mass, $m_{g,p}$ is the pre-clutch gearing(s) mass (es), $m_{c}$ is the clutch(s) mass (es), $m_{g,m}$ is the main gearing(s) mass (es), $m_{g,r}$ is the recombination gearing mass and $m_{f}$ is the frame mass. The mass of each component is defined by Equations 23–28:

$$m_{m}=\frac{T_{m}}{\tau_{m,c}\left(OL_{m}\right)}$$ (23)

$$m_{g,p}=\frac{T_{c}}{\tau_{g,p,c}\left(OL_{g}\right)}$$ (24)

$$m_{c}=\frac{T_{c}}{\tau_{c}}$$ (25)

$$m_{g,m}=\frac{T_{a,ind}}{r_{r}\tau_{g,m,c}\left(OL_{g}\right)N}$$ (26)

$$m_{g,r}=\frac{T_{a,ind}}{\tau_{g,r,c}\left(OL_{g}\right)}$$ (27)

$$m_{f}=\frac{T_{a,ind}}{\tau_{f,c}\left(OL_{f}\right)}$$ (28)

where $\tau_{m,c}$ , $\tau_{g,p,c}$ , $\tau_{c}$ , $\tau_{g,m,c}$ , $\tau_{g,rc}$ , $\tau_{f,c}$ are the components rated continuous torque densities and $OL_{g}$ , $OL_{f}$ are gearing and frame overload factors similarly as the motor overload factor $\left(OL_{m}\right)$ of Equation 15. Design overload factors are kept explicit in the model to later compare designs with varying levels of aggressiveness which could translate into component optimization and costs. The concept of overload is not used for MR clutches since they are designed such that their rated torque is obtained at magnetic saturation and thus, they can’t be significantly overloaded beyond their rated maximum torque or otherwise they slip. Assuming that the frame and gears have an identical overload factor $\left(OL_{f}=OL_{g}\right)$ , then:

$$\frac{T_{a,ind}}{m_{a}}=\frac{i}{\frac{1}{r_{p}\tau_{m,c}\left(OL_{m}\right)}+\frac{1}{\tau_{g,p,c}\left(OL_{g}\right)}+\frac{1}{\tau_{c}}+\left(\frac{1}{r_{r}\tau_{g,m,c}}+\frac{1}{\tau_{g,r,c}}+\frac{1}{\tau_{f,c}}\right)\frac{i}{OL_{g}}}.$$ (29)

Equation 29 holds for conventional actuators as well by setting $\tau_{c}=\tau_{g,p,c}=\tau_{g,m,c}=\infty$ . Parameter values for calculations are listed in Table 1 (Plante et al., 2025).

**TABLE 1:** Data for the calculation of the torque-to-mass ratio.

Parameters	Units	Single Path (Gen1)	Multiple Path (Gen2)	Multiple Path (Gen3)	Harmonic- drive (UR)
$\tau_{m,c,ref}$	Nm/kg	1.14	1.14	4.00	0.91
$T_{m,c,ref}$	Nm	0.17	0.17	0.54	2.70
$\tau_{c,ref}$	Nm/kg	5.92	6.47	10.00	∞
$T_{c,ref}$	Nm	0.80	1.20	1.20	—
$\tau_{g,p,c}$	Nm/kg	46.00	46.00	∞	∞
$\tau_{g,m,c}$	Nm/kg	56.70	68.20	68.20	∞
$\tau_{g,r,c}$	Nm/kg	138.30	414.50	414.50	83.70
$\tau_{f,c}$	Nm/kg	128.00	365.00	365.00	365.00
$OL_{m}$	—	1.00	1.81	2.60	0.93
$OL_{g}$	—	1.00	1.00	2.00	2.35
$r_{p}$	—	4.80	3.96	1.00	1.00
$r_{r}$	—	4.80	7.18	7.18	100.00
N	—	2	2	2	1

Overload factors for harmonic drives are estimated from component manufacturers data while those of geared MR actuator designs were selected based on growing confidence as prototype generations evolve. The first generation used a conservative OL of 1 while latest generations used OL similar to those of harmonic drive gearing. Motor overload factors of 2–3 are used as generally recommended by motor manufacturers.

**FIGURE 4:**
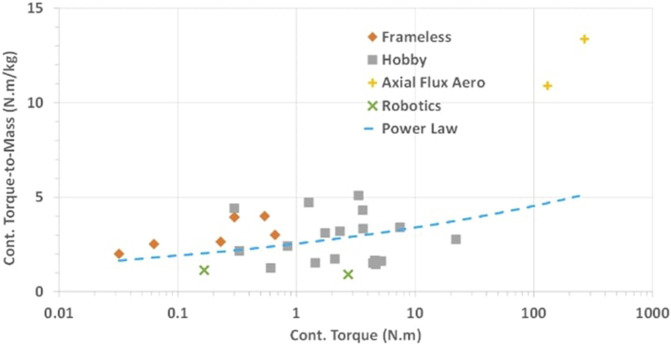
Motor torque-to-mass vs. motor torque data extracted from various commercially available motors. Adapted from Plante et al. (2025).

**FIGURE 5:**
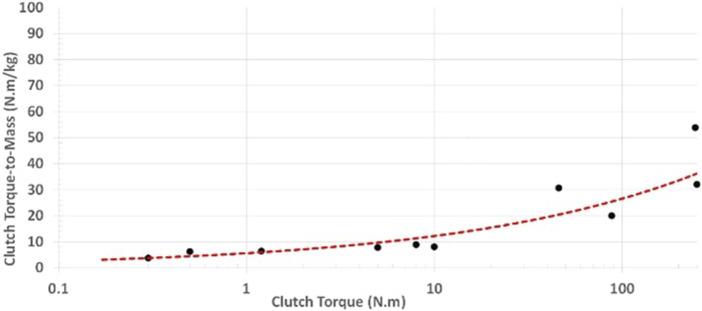
MR clutch mass vs. clutch torque data extracted from various MR clutch designs built over the years. Adapted from Plante et al. (2025).

Motor and clutch torque-to-mass vary non-linearly with size as shown in Figures 4, 5, such that they can be defined by Equations 30, 31:

$$\tau_{m,c}=2.53{\left(T_{m,c}\right)}^{0.13}$$ (30)

$$\tau_{c,drum}=5.61{\left(T_{c}\right)}^{0.34}$$ (31)

where $\tau_{c,drum}$ is the drum-type clutch torque-to-mass. The data on Figure 4 shows a large scatter since it includes widely varying motor architectures (e.g., actively cooled axial flux vs. classical robotics motors). The fit is a first order model intended to represent a general trend of selected relevant motor technologies. Equation 32 holds when scaling from a known reference motor size:

$$\tau_{m,c}=\tau_{m,c,ref}{\left(\frac{T_{m,c}}{T_{m,c,ref}}\right)}^{0.13}.$$ (32)

Disk clutches as in the *Gen3* have not been studied as much as drum clutches. First measurements on a 3D printed disk clutch prototype showed twice the torque density of the drum clutches of the *Gen2* generation (Lhommeau et al., 2023). Although the torque-to-mass of both disk and drum clutches could be further optimized, a conservative torque density boundary is set for disk clutches as a fraction of drum clutches such that $\tau_{c,disk}=1.5\tau_{c,drum}$ . A single scaling law for any clutch design (disk or drum) is then derived (see Equation 33) by dividing Equation 31 by itself:

$$\tau_{c}=\tau_{c,ref}{\left(\frac{T_{c}}{T_{c,ref}}\right)}^{0.34}.$$ (33)

### 2.5 Actuator torque-to-inertia

Geared MR actuator output inertia is the sum of the clutches and gearing reflected inertia, $I_{c,out}$ and $I_{g,out}$ , respectively (see Equation 34):

$$I_{a}=NI_{c,out}+NI_{g,out}.$$ (34)

The torque-to-inertia ratio is then defined by Equation 35:

$$\frac{T_{a,ind}}{I_{a}}=\frac{NT_{c}i}{NI_{c,out}+NI_{g,out}}$$ (35)

which can be rewritten as Equation 36:

$$\frac{T_{a,ind}}{I_{a}}=\frac{T_{c}}{I_{c,out}}\left(\frac{i}{1+\frac{I_{g,out}}{I_{c,out}}}\right)=\frac{\alpha_{c}}{i}\left(\frac{1}{1+\frac{I_{g,out}}{I_{c,out}}}\right)$$ (36)

where $\alpha_{c}=\left(T_{c}/I_{c}\right)$ is the clutch torque-to-inertia ratio. An expression for $\left(I_{g,out}/I_{c,out}\right)$ is now derived based on experimentally validated scaling laws for robotic transmission systems (Saerens et al., 2019). Starting with clutch inertia, it is assumed linearly proportional to clutch torque. The reflected clutch inertia at output is thus defined by Equation 37:

$$I_{c,out}=\frac{1}{\alpha_{c}}T_{c}i^{2}N$$ (37)

Now, the inertia of planetary and parallel shaft (spur) gears scales with $I_{g}\propto \left(Ld^{4}i^{2}\right)/a$ (Saerens et al., 2019) , giving Equation 38:

$$I_{g}=0.5kMd^{2}i^{2}$$ (38)

where $M=\left(\rho \pi Ld^{2}\right)/a$ is the gearing mass, d the diameter, a the number of stage, and k a constant specific to the gearing design (e.g., spur, planetary, harmonic, etc.). The gearing mass can be expressed in terms of the torque density, as shown in Equation 39:

$$M=\frac{\tau_{g,c}OL_{g}}{T_{g}}$$ (39)

where $T_{g}$ is the gearing rated torque, $\tau_{g,c}$ the gearing continuous torque-to-mass ratio and $OL_{g}$ the gearing overload factor. Eliminating the gearing mass in Equation 38 gives Equation 40:

$$I_{g,out}=\frac{0.5kT_{g}d^{2}i^{2}}{\tau_{g,c}OL_{g}}.$$ (40)

In geared MR actuators, the majority of gearing inertia comes from the main gearing stage. Applying Equation 40 to the main gearing stage gives Equation 41:

$$I_{g,out}=\frac{0.5kT_{c}id^{2}{\left(r_{m}\right)}^{2}{\left(r_{r}\right)}^{2}N}{\tau_{g,m,c}OL_{g}}.$$ (41)

The gearing-to-clutch inertia ratio is then defined by Equation 42:

$$\frac{I_{g,out}}{I_{c,out}}=\frac{0.5k\alpha_{c}id^{2}}{\tau_{g,m,c}OL_{g}r_{r}}.$$ (42)

Substituting back into Equation 36 gives Equation 43:

$$\frac{T_{a,ind}}{I_{a}}=\frac{1}{\frac{i}{\alpha_{c}}+\frac{0.5kd^{2}i^{2}}{\tau_{g,m,c}OL_{g}r_{r}}}.$$ (43)

Gearing diameter can be expressed in non-geometrical parameters to eliminate all dimensionality. Since gearing torque scales with volume (Saerens et al., 2019), the main gearing torque scaling law with volume is defined with Equation 44:

$$\frac{T_{a,ind}}{Nr_{r}}\propto \frac{Ld^{2}}{a}.$$ (44)

Finding an expression between gearing length and diameter would eliminate all dimensional quantities from Equation 43, simplifying the analysis. Inspecting manufacturers data (for example, compare Harmonic Drive’s planetary drives HPG size 20 and size 50) suggests that it is reasonable, at least in first approximation, to assume that gearing length is roughly proportional to output torque with a power of 0.3. This means that a ten fold increase in torque doubles the length such that:

$$L\propto {\left(\frac{T_{a,ind}}{Nr_{r}}\right)}^{0.3}.$$ (45)

Equation 45 is assumed to hold for all gearing type, harmonic or planetary. In any case, it has been verified that the torque to length relation of Equation 45 has a second order effect on model predictions and does not change conclusion of the work. Then, Equation 46:

$$d^{2}\propto a{\left(\frac{T_{a,ind}}{Nr_{r}}\right)}^{0.7}$$ (46)

allows to obtain Equation 47:

$$\frac{T_{a,ind}}{I_{a}}=\frac{1}{\frac{i}{\alpha_{c}}+\frac{k{\left(T_{a,ind}\right)}^{0.7}i^{2}}{\tau_{g,m,c}OL_{g}N^{0.7}{\left(r_{r}\right)}^{1.7}}}.$$ (47)

Equation 43 shows that minimizing gearing diameter is critical for high torque-to-inertia, which, as shown by Equation 47, is done by using multiple actuation chains (N>1) and high recombination gearing $\left(r_{r}>1\right)$ . Values for calculations are listed in Table 2.

**TABLE 2:** Data for the calculation of the torque-to-inertia ratio.

Parameters	Units	Single Path (Gen1)	Multiple Path (Gen2)	Multiple Path (Gen3)	Harmonic- drive (UR)
$\alpha_{\mathrm{ref}}=\alpha_{m}$	$\frac{Nm}{kg.m^{2}}$	—	—	—	28.977
$T_{\mathrm{ref}}=T_{m}$	Nm	—	—	—	2.70
k	—	—	—	—	$1.7\times 10^{-6}$
$\alpha_{\mathrm{ref}}=\alpha_{c}$	$\frac{Nm}{kg.m^{2}}$	$1.5\times 10^{6}$	$1.7\times 10^{6}$	$5.3\times 10^{6}$	—
$T_{\mathrm{ref}}=T_{c}$	Nm	0.8	1.2	1.2	—
k	—	$4.6\times 10^{-6}$	$2.5\times 10^{-7}$	$6.3\times 10^{-8}$	—
$\tau_{g,m,c}$	$\frac{Nm}{kg.m}$	56.7	68.2	68.2	—
$OL_{g}$	Nm	1.0	1.0	2.3	—
$\tau_{g,r,c}$	$\frac{Nm}{kg.m}$	—	—	—	83.7
$OL_{g}$	Nm	—	—	—	2.3
$r_{r}$	—	4.80	7.18	7.18	100.00
N	—	2	2	2	—

For conventional actuators, MR clutch properties are simply replaced by motor properties in Equations 43 or 47. Also, gearing reduction is entirely done in the recombination gearing stage such that a=1 , N=1 , $r_{r}=1$ and $\tau_{g,m,c}$ is replaced with $\tau_{g,r,c}$ , leading to Equation 48:

$$\frac{T_{a,ind}}{I_{a}}=\frac{1}{\frac{i}{\alpha_{m}}+\frac{k{\left(T_{a,ind}\right)}^{0.7}i^{2}}{\tau_{g,r,c}OL_{g}}}.$$ (48)

Equations 47, 48 show that the main design parameters are related to *actuator size* (torque output $T_{a,ind}$ ), *clutch or motor quality* (torque-to-inertia ratio $\alpha_{c}$ or $\alpha_{m}$ ), *gearing quality* (torque density $\tau_{g,m,c}$ or $\tau_{g,r,c}$ and $OL_{g}$ ) and *gearing topology* (gearing ratio i , number of chain N and recombination ratio $r_{r}$ ).

Finally, the inertia correction factor k is fitted by back-calculating over known actuator data. For example, for a geared MR actuator, k is given by Equation 49:

$$k=\frac{\tau_{g,m,c}OL_{g}N^{0.7}{\left(r_{r}\right)}^{1.7}}{{\left(T_{a,ind}\right)}^{0.7}i^{2}}\left(\frac{1}{\frac{T_{a,ind}}{I_{a}}-\frac{i}{\alpha_{c}}}\right).$$ (49)

The correction factor k is assumed constant for all actuators of a same design family.

Here again, motor and drum-type clutch torque-to-inertia vary non-linearly with size. The torque-to-inertia $\zeta_{c}$ of drum clutches vary with torque, as shown in Equation 50:

$$\frac{\zeta_{c,2}}{\zeta_{c,1}}={\left(\frac{T_{c,2}}{T_{c,1}}\right)}^{-0.806}.$$ (50)

If $\zeta_{c,1}$ and $T_{c,1}$ are a known reference clutch $\zeta_{c,ref}$ and $T_{c,ref}$ , then the torque-to-inertia of any similar clutch of different size is defined by Equation 51:

$$\zeta_{c,2}=\zeta_{c,ref}{\left(\frac{T_{c,2}}{T_{c,ref}}\right)}^{-0.806}.$$ (51)

Since $T_{c,2}=T_{a,ind}/\left(Ni_{2}\right)$ , a general relationship for geared MR actuator torque-to-inertia can be written as Equation 52 (Plante et al., 2025):

$$\zeta_{a,ind}=\frac{1}{\frac{i^{0.194}}{\zeta_{\mathrm{ref}}}{\left(\frac{T_{a,ind}}{T_{\mathrm{ref}}N}\right)}^{0.806}+\frac{k{\left(T_{a,ind}\right)}^{0.7}i^{2}}{\tau_{g,m,c}OL_{g}N^{0.7}{\left(r_{r}\right)}^{1.7}}}.$$ (52)

The scaling law of Equation 50 is assumed to hold for disk MR clutches and for electric motors since they are all similar electromechanical machines. A general relationship for conventional actuators is then defined by Equation 53:

$$\zeta_{a,ind}=\frac{1}{\frac{i^{0.194}}{\zeta_{\mathrm{ref}}}{\left(\frac{T_{a,ind}}{T_{\mathrm{ref}}}\right)}^{0.806}+\frac{k{\left(T_{a,ind}\right)}^{0.7}i^{2}}{\tau_{g,r,c}OL_{g}}}.$$ (53)

### 2.6 Experimental validation

Collision tests are carried out on an *Exonetik* MR5 cobot to verify the *Torque-to-Mass* and *Torque-to-Inertia* ratios as well as the collision model accuracy. The MR5 is a development robot not available commercially. The MR5 shares the UR5e architecture, payload and workspace but uses MR actuators with a maximum joint torque of 140 Nm in combined slip mode. The MR actuators are *Gen2* designs with drums-type clutches and multiple loadpath gearing.

**FIGURE 6:**
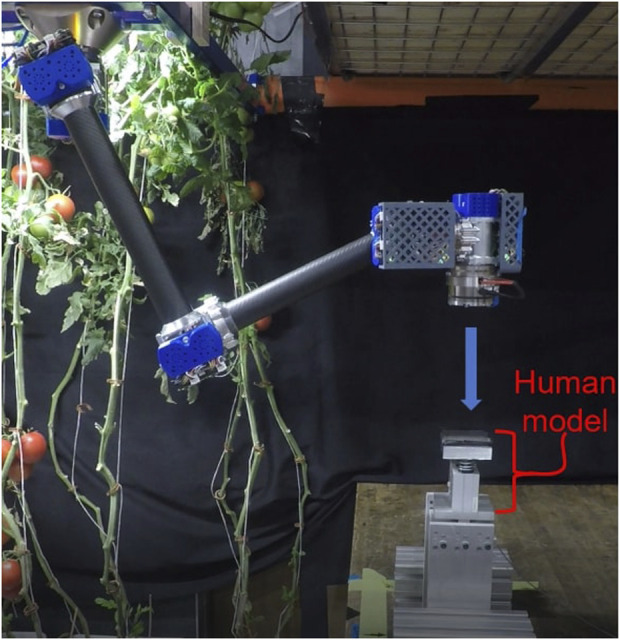
Experimental setup of the collision tests carried out on the MR actuated cobot MR5.

The experiments are based on those carried in previous work (St-Jean et al., 2024) using the same test bench. They consist of first positioning the robots with a known joint configuration, far from kinematic singularities; then commanding a linear trajectory to reach a collision point at a second joint configuration with a known collision speed. The trajectory is determined to reach a collision perpendicular to the surface such as in the ISO/TS 15066 Standard. A uniaxial force sensor under the mass-spring (0.6 kg, 75 N/mm) representing a human at the collision point allows to measure contact forces. The experimental setup is presented in Figure 6.

**FIGURE 7:**
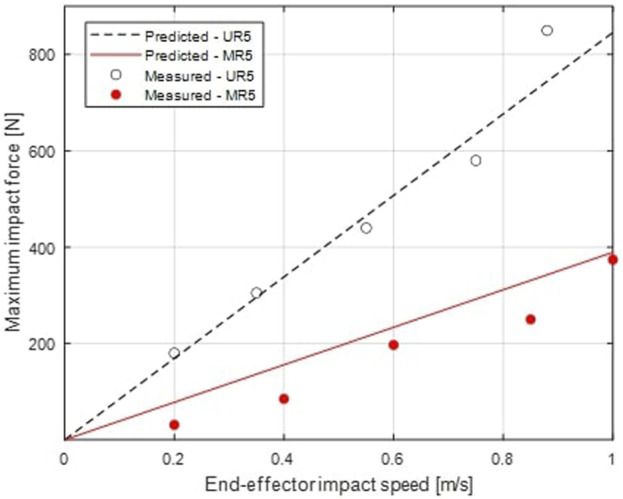
Measured maximum impact force from a collision test compared to the model prediction, as a function of end-effector impact speed, for both UR5 and MR5 cobots.

Measured impact forces are presented in Figure 7 alongside model predictions. The collision test results carried out by St-Jean (St-Jean et al., 2024) on the UR5 are also compared in Figure 7 to the model predictions. Both robots show good fit between the predictions and the experimental values, even considering the multiple assumptions made in the actuator mass and inertia models, with $R^{2}=0.944$ for the MR5 collision and $R^{2}=0.973$ for the UR5 collision, validating the model fidelity. Poorer fit for the MR5 is mainly due to the unconsidered active and passive joint stiffness in the prediction model, which benefits higher impact forces.

### 2.7 Robot reflected mass performance metric

A performance metric must be derived to evaluate the general safety of a robot over its entire workspace to compare various robot designs. Many metrics from the literature have been considered such as the reflected mass of a constant operational direction, the Mean Reflected Mass (MRM) (Steinecker et al., 2022), the Generalized Impact Measure (GIM) (Walker, 1994) and the volume of the mass ellipsoid (Song et al., 2020). The volume of the mass ellipsoid, which defines a reflected mass for every directional vector, represents the overall impact strength, but cannot directly transpose to an effector speed and could lead to false impressions of safety when the ellipsoid is skewed with a specific direction having a huge reflected mass despite a small ellipsoid volume. The GIM expresses the difficulty for a human to move the end-effector along a trajectory but is devoid of any physical interpretation (Steinecker et al., 2022). The MRM optimization showed a correlation with lower forces in collision, but lacks an analytical expression, hence has unclear mathematical relationship with the robot configuration (Steinecker et al., 2022).

The choice is made to use the median of the maximal reflected masses of numerous robot’s poses over its workspace, described here as the *Global maximal reflected mass* $M_{gm}$ . The motivation is that the maximal reflected mass for a given pose represents the worst collision case possible for this pose and is easier to transpose to an equivalent “safe speed” at the robot end-effector. The maximal reflected mass is the principal axis of the mass ellipsoid and is associated with the eigenvector of Λ(x) (Lynch and Park, 2016). Also, the median is preferred as the maximal reflected mass distribution throughout the useful workspace does not follow a normal law as there are aberrant values near singularities. The median allows to ignore aberrant values and to find a $M_{gm}$ value that is closer to reality since most robot operators simply avoid singularities anyway.

It should be noted that some of the metrics discussed above were implemented and compared to the $M_{gm}$ metric and gave similar results and trends, giving confidence in the validity of the metric and showing that, ultimately, the choice of the metric is not so critical to the validity of the conclusions of this work.

In this study, we limit ourselves to robot architectures identical to, or derived from UR robots because, as explained before, they represent a well known gold standard reference.

Robot joint configurations selected for $M_{gm}$ computation are determined by a filtered Latin Hypercube Sample (LHS) to uniformly represent the workspace. The filter applied to the LHS allows the sample to stay away from singularities and is based on the Jacobian determinant of each pose having an absolute value equal or higher than 0.005, as singularities are found when the Jacobian determinant gets close to zero. This threshold filters the ∼20% worst poses for path planning for a UR5e architecture. The LHS does not consider the base joint since it has negligible influence on the reflected mass for a UR type robot architecture. The UR architecture also allows to bound the LHS for the second joint between 0° and 90° as the robot is symmetrical otherwise. The last joint is excluded as well from the LHS because it has no effect on the reflected mass for this study, as it is considered that the effector is positioned on the axis of this joint and only translational collisions are considered. It has been verified that the used LHS sample of about 115,000 poses after filtering gives the same $M_{gm}$ as 700,000 poses within a tolerance of ±0.43% , meaning bigger samples have small to no effect on the results.

In all, unless otherwise noted, all optimizations run in this paper aim to find the optimal global gearing ratio $R_{\mathrm{opt}}$ minimizing $M_{gm}$ when applied to all 6 actuators. Bounds are established to keep gearing ratios within practical limits of [5–200] for MR actuators and [50–160] for HD actuators. Values beyond these ranges have been tested without yielding improved results.

Four analyses are performed: (1) a global gearing ratio i optimization is first carried out on the UR5e robot from Universal Robots. (2) Then, a more in-depth analysis is performed by optimizing hypothetical UR5e robots with different reaches while respecting UR5e proportions. (3) Another hypothetical robot is optimized around the UR5e by delocalizing the last three actuators of the wrist down to the elbow. (4) Finally, the effect of optimizing each actuator gearing ratio individually is observed through an optimization on the UR5e. To do so, multiple gradient descents are done using Matlab “fmincon” and “MultiStart” functions to obtain an optimal combination of 6 gearing ratios, one for each actuator.

## 3 Results

Results are expressed in terms of reflected masses at end-effector. When relevant, the reflected masses are translated to maximum safe speeds according to ISO/TS 15066 Standard (ISO, 2016). Also, it is worth recalling that the global gearing ratio i used everywhere in the results section refers to the combination of the main gearing stage, $r_{m}$ , and the recombination gearing stage, $r_{r}$ , for a total actuator gearing of $i=r_{m}r_{r}$ .

### 3.1 Influence of gearing ratio on $M_{gm}$

**FIGURE 8:**
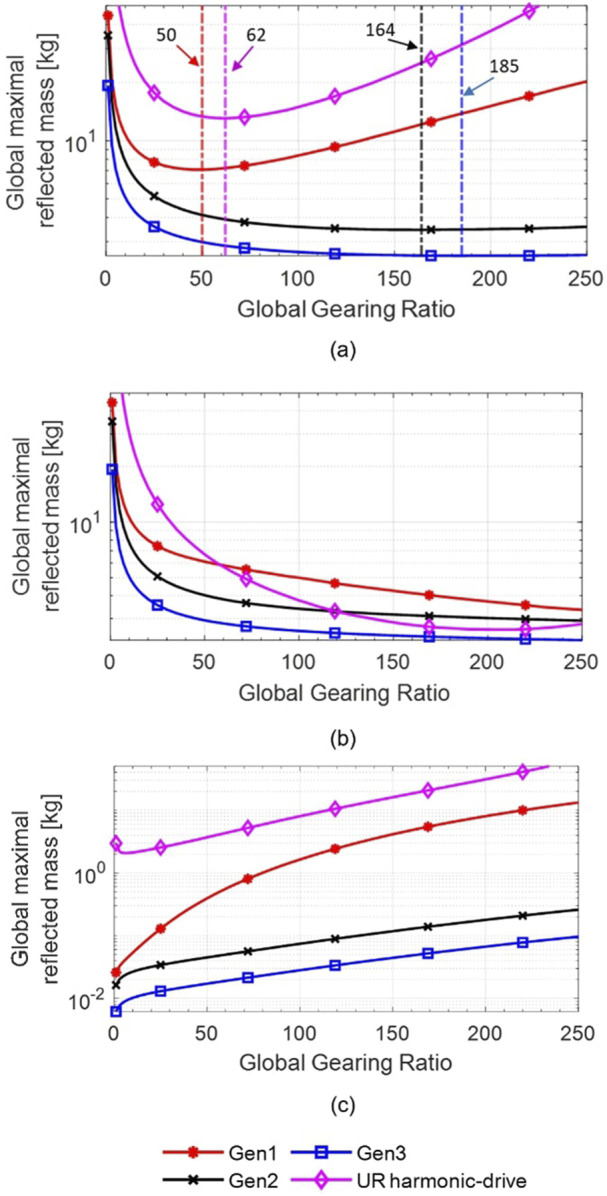
Results from the analytical model for the global maximal reflected mass $\left(M_{gm}\right)$ based on the global gearing ratio on a UR5e for the 4 studied actuators considering: **(a)** Structural inertia matrix $A_{s}$ and Actuator’s inertia matrix $A_{a}$ , **(b)** Structural inertia matrix $A_{s}$ only, and **(c)** Actuator’s inertia matrix $A_{a}$ only. Dotted lines on **(a)** are positionned at the minimum $\left(M_{gm}\right)$ for each curve.

The effect of gearing ratio on $M_{gm}$ for cobots using a UR5e geometry is shown in Figure 8 for the 4 studied actuators. Figure 8a represents the actual $M_{gm}$ , as Figures 8b,c represent $M_{gm}$ as if only the *Structural inertia matrix* $A_{s}$ and only the *Actuator’s inertia matrix* $A_{a}$ are considered in the calculation of $M_{gm}$ , respectively.

Figure 8b shows the reduction of the reflected mass associated with the reduction of the structural mass as the gearing ratio increases, while Figure 8c shows the increase of the reflected mass associated with the increase of actuator’s output inertia following the gearing ratio increase. These opposing trends create the presence of $R_{\mathrm{opt}}$ , as seen by the minimum on both curves for *Gen1* and HD actuators (50:1 for *Gen1*, 62:1 for HD) on Figure 8a. *Gen2* and *Gen3* actuators show different results as the curves flatten above 100:1. Thus, the optimal gearing ratios (164:1 for *Gen2*, 185:1 for *Gen3*) are not as visible. This situation is explained by Figure 8c where the actuator’s inertial reflected masses of *Gen2* and *Gen3* remain small enough to be negligible while they increase significantly for *Gen1* and HD. A vertical dotted line is drawn at each actuator’s *minimum Global reflected mass* to ease the reading of Figure 8a.

**FIGURE 9:**
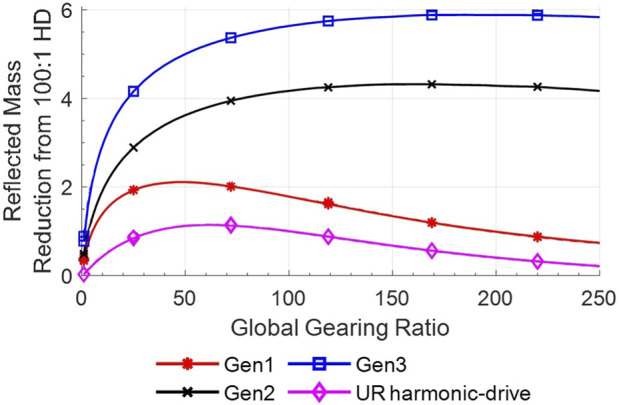
Results from the analytical model for the reduction ratio of the reflected mass $\left(R_{\mathrm{100:1,HD}}\right)$ based on the global gearing ratio on a UR5e from the use of UR Harmonic-drive actuators with a 100:1 gearing ratio compared with the use of MR actuators (Gen1, Gen2 and Gen3) and UR Harmonic-drive actuators with a different gearing ratio.

The reduction of the reflected mass between the studied actuators and 100:1 HD actuators, $R_{\mathrm{100:1,HD}}$ , is shown in Figure 9 and is defined by Equation 54:

$$R_{100:1,HD}=\frac{M_{gm,100:1,HD}}{M_{gm}}$$ (54)

where $M_{gm,100:1,HD}$ is $M_{gm}$ from the use of 100:1 HD actuators.

The improvement factors of Figure 9 suggest that MR actuators have the potential to reduce the reflected mass by a factor ranging from ∼ 2X to ∼ 6X depending on configurations. At the time of writing, the *Gen2* actuator design with a gearing ratio of 57:1 has been demonstrated experimentally in the MR5 robot and shows a reflected mass reduction of ∼ 4X. The highest possible reduction is seen for *Gen3* actuators, yet theoretical, at a gearing of 185:1 and would result in an increase of the maximal safe speed at the end-effector of ∼ 2X.

It should be noted that the maximum actuator safe speed associated with the calculated optimal $M_{gm}$ is not always achievable because of motor speed limitations. This is mostly observed with higher optimal gearing ratios such as *Gen2* and *Gen3*. For example, *Gen2* actuators using Maxon motors and optimal gearing of 164:1 can only reach their maximal safe speeds in 7% of the studied poses. In contrast, *Gen3* actuators using TQ frameless brushless motors and an optimal gearing of 185:1 can reach their maximal safe speeds in 66% of the poses. As shown in Figure 9, the penalty of reducing the gearing ratio in order to reach higher speeds is almost negligible for *Gen2* and *Gen3* until 100:1 due to the plateaued shape of the performance curves.

### 3.2 Influence of robot reach on $M_{gm}$

**FIGURE 10:**
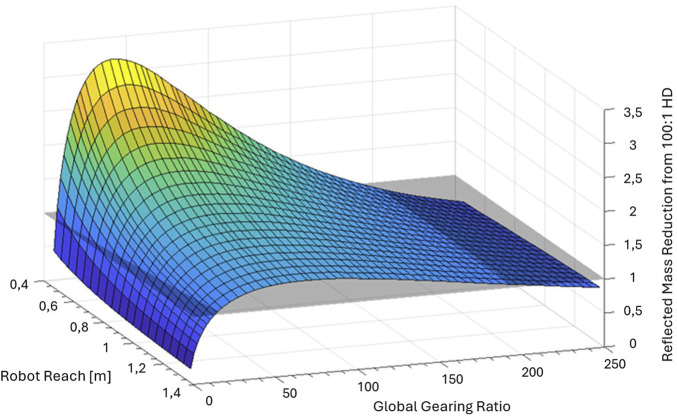
Results from the analytical model for the reflected mass reduction from the use of Gen1 actuators instead of 100:1 Harmonic-Drive actuators $\left(R_{\mathrm{100:1,HD}}\right)$ based on the robot reach.

The effect of varying robot reach and gearing on a map of $R_{\mathrm{100:1,HD}}$ functions is shown on Figure 10. Again, the base architecture for scaling is a UR5e robot. Actuator sizes are kept constant to UR5e actuator sizes for all scenarios. The bounds of the analysis are between the UR3e reach (0.5 m) and the UR10e reach (1.3 m). Results consider *Gen1* actuators, but other MR actuators show the same pattern. The map shows that the $R_{\mathrm{100:1,HD}}$ functions are stretched-up when the robot reach is lower, and flatten when higher. As the robot reach increases, actuators’ masses get farther from the base and their rotation point, increasing the structural inertia that gradually becomes dominant over actuator inertia. Hence for given actuator torques, shorter robots have a greater potential of reflected mass reduction from the gearing ratio optimization of MR actuators than robots with longer reach.

### 3.3 Influence of delocalization on $M_{gm}$

**FIGURE 11:**
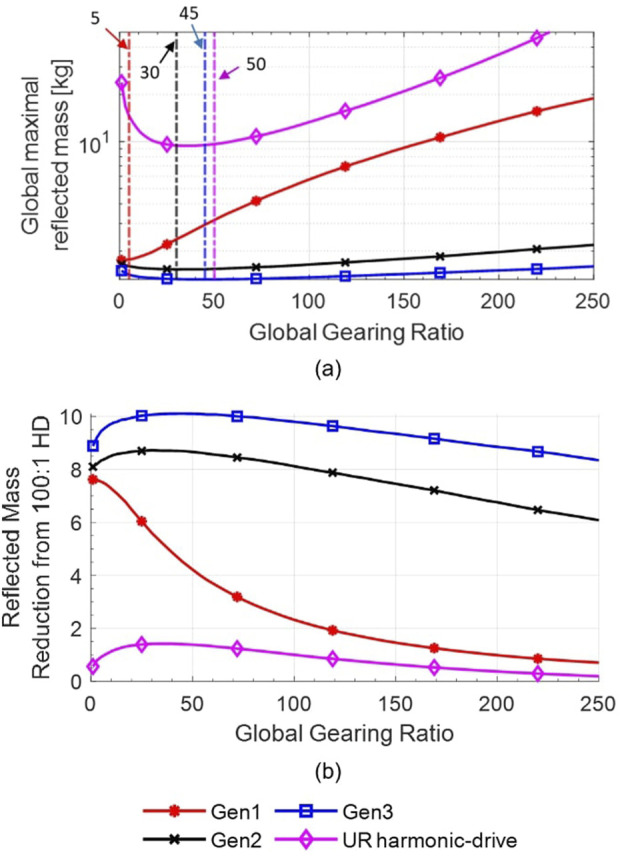
Results from the analytical model for the **(a)** Global maximal reflected mass $\left(M_{gm}\right)$ and **(b)** Reflected mass reduction from 100:1 HD actuators $\left(R_{\mathrm{100:1,HD}}\right)$ , on an UR5e with wrist actuators delocalized to the elbow.

The effect of delocalizing wrist actuators to the elbow of a UR5e robot is shown on Figure 11. Figure 11a is similar to Figure 8c, but $R_{\mathrm{opt}}$ are lower for all designs. The reduced distance between the three wrist actuators and the robot base reduces the influence of those actuators’ mass on $A_{s}$ from Equation 11, similarly to the shorter reach studied earlier. Thus, heavier actuators with lower inertia become more profitable to reduce the reflected mass. As a result, optimal gearing ratios for cobots with delocalized wrist actuators is toward the lower bound of the gearing ratio range as $M_{gm}$ becomes almost entirely driven by $A_{a}$ . This statement assumes that the added mass from the delocalizing transmission is negligible, for example, if using cables. As seen on Figure 8a, a vertical dotted line is drawn at each actuator’s *minimum Global reflected mass* to ease the reading of Figure 11a. Figure 11b presents $R_{\mathrm{100:1,HD}}$ for the delocalized robot, where *Gen3* shows ∼ 10X improvement from the UR5e with delocalized 100:1 HD actuators, compared to the ∼ 6X achieved with the non-delocalized actuators.

### 3.4 Influence of multiple gearing ratios on $M_{gm}$

So far in this paper, actuators of a given robot configuration all used the same gearing ratio. Theoretically, further reflected mass reductions are possible if each joint/actuator has it’s own gearing ratio. The optimal gearing ratios of the actuators studied in this paper when used on a UR5e configuration are shown in Table 3.

**TABLE 3:** Results from the analytical model for the optimal gearing ratios from the multiple gearing ratios $M_{gm}$ Optimization.

Actuators	Optimal gearing ratios					
	Base	Shoulder	Elbow	Wrist	Wrist	Wrist
Harmonic-Drive	50	50	54	76	90	160
Gen1	5	36	30	54	63	200
Gen2	5	132	103	185	187	200
Gen3	5	133	117	182	198	200

Table 4 shows the comparison of $R_{\mathrm{100:1,HD}}$ for the single and multiple gearing ratios optimizations on the UR5e. Results show that differences between uniform and individual ratios are small. As the complexity and cost for having distinct gearing ratios at every actuator would likely increase, using a unique gearing ratio for all actuators appears sufficient.

**TABLE 4:** $R_{\mathrm{100:1,HD}}$ comparison between a single optimal gearing ratio and multiple optimal gearing ratios on the UR5e.

Actuators	$R_{\mathrm{100:1,HD}}$ Same gearing	$R_{\mathrm{100:1,HD}}$ Individual gearings
Harmonic-Drive	1.14	1.24
Gen1	2.11	2.27
Gen2	4.32	4.44
Gen3	5.89	5.98

## 4 Discussion

### 4.1 Design guidelines

The work gave a first description of the design space of geared MR actuators used in serial-chain cobots by studying how much gearing can be used between the MR actuators and the joint outputs while remaining safe to humans.

The following design guidelines can be drawn:

Design guideline 1: From a safety standpoint according to ISO/TS 15066 Standard, MR actuators have potential of producing a lower Global maximal reflected mass $\left(M_{gm}\right)$ , over a wide range of gearing ratios, up to at least 200:1, leading to faster safe speeds with reduced actuator mass. MR actuators showed a potential of reducing $M_{gm}$ by a factor of ∼ 2X (with the *Gen1*) to ∼ 6X (with the *Gen3*) when using optimal gearing ratios, providing up to 2.14X faster safe operational speed than the standard UR5e with 100:1 HD actuators. In all, it can be said that, within the limits of this study, geared MR actuators have the potential to roughly double the safe speed of UR5-type robots. As shown in (St-Jean et al., 2024), such better performing passive systems are synergistic to the use of active strategies, like collision detection and braking which can further increase safe speeds.

Design guideline 2: Optimal gearing ratios are highly dependent on MR actuator torque and inertia densities. From the generations analyzed in this study, the *Gen1* actuator (single load-path, drum-clutch) presents a clear gearing ratio optimum value around 50:1, while the better performing *Gen2* (multiple load-path, drum-clutch) and *Gen3* actuators (multiple load-path, disk-clutch) have no optimal values within the 200:1 gearing ratio bound used in this study. The implication is that geared MR actuator must reach a certain threshold level of torque/inertia density to use high gearing ratios. With the current state of technology, this necessitates the use of multiple shear interfaces in the MR clutches (e.g., multi-disk or multi-drum configurations) and multiple contact points gearing (e.g., 3 planets planetary gearing).

Design guideline 3: For given actuator torques, the performance improvement potential of MR actuators decreases with robot size or reach. Indeed, smaller robots with smaller reach better exploit the advantages of MR actuators since most of their reflected mass comes from actuator rotational inertia. As robot size increases, reflected mass shifts to structural rather than inertial mass.

Design guideline 4: Actuator delocalization such as bringing the wrist actuators to the elbow removes structural mass and thus shifts the optimal global gearing ratio to lower ratios. With more emphasis on inertial mass, the performance potential of MR actuators also increases, and the studied delocalized robot showed a $M_{gm}$ reduction of up to 10X from the use of *Gen3* actuators instead of the 100:1 HD actuators.

Design guideline 5: Optimizing a serial chain robot with a distinct gearing ratio for each actuator does not significantly reduce $M_{gm}$ . Unless considering highly-specialized application with optimized tasks, the added complexity and cost of having distinct actuator designs at each joint is not worth the safety/speed benefit.

### 4.2 Limitations

Results of this paper should be used with care since it is only a first step toward understanding the design space of MR-powered robots. Fully understanding the design space of a technology with extensive experimental and analytical results covering all possible designs is a massive effort that cannot be done in a single research paper. Accordingly, the work presented here has important limitations, with two important ones being:

1- The analytical model of the geared MR actuator mass and inertia has many simplifications that were necessary to extract workable closed-form analytical formulations. The model is assumed valid over the range of its experimental calibration, here limited to three similar actuator designs (Gen 1,2,3). Model validity is unknown past its calibration range, for example, when considering widely varying geared MR actuator designs.

2- The study is limited to a single 6 DOF robot architecture based on the UR5e and its harmonic drive actuator design. The conclusions of this paper are only deemed valid for the UR5e geometry and may not apply to other robot and/or actuator designs.

### 4.3 Future works

Future works should improve the richness of knowledge by studying different geared MR actuator designs in different robot geometries to bring new data points. Further investigations should also be done toward other critical robot metrics regarding the effect of gearing ratio on stiffness, bandwidth, backdrivability, disturbance rejection, etc. At last, more collision data of 6 DOF robots using various MR actuator designs are needed to fully validate the proposed collision model over the full workspace.

## Data Availability

The raw data supporting the conclusions of this article will be made available by the authors, without undue reservation.
